# The Effectiveness of Video Animations as a Tool to Improve Health Information Recall for Patients: Systematic Review

**DOI:** 10.2196/58306

**Published:** 2024-12-30

**Authors:** Steffen Hansen, Tue Secher Jensen, Anne Mette Schmidt, Janni Strøm, Peter Vistisen, Mette Terp Høybye

**Affiliations:** 1 University Clinic for Interdisciplinary Orthopedic Pathways (UCOP) Elective Surgery Center Silkeborg Regional Hospital Silkeborg Denmark; 2 Diagnostic Imaging Silkeborg Regional Hospital Silkeborg Denmark; 3 Department of Sport Science and Clinical Biomechanics University of Southern Denmark Odense Denmark; 4 University Clinic for Innovative Patient Pathways Medical Diagnostic Centre Silkeborg Regional Hospital Silkeborg Denmark; 5 University Research Clinic for Innovative Patient Pathways Medical Diagnostic Centre Silkeborg Regional Hospital Silkeborg Denmark; 6 Quality Section Capio Private Hospitals Viborg Denmark; 7 Department of Communication & Psychology Aalborg University Aalborg Denmark; 8 Interacting Minds Centre Department of Clinical Medicine Aarhus University Aarhus Denmark

**Keywords:** public health, health information, patient information, animation video, digital health, visualization, memory, recall, education, synthesis, review methods, review methodology, systematic, PRISMA

## Abstract

**Background:**

Access to clear and comprehensible health information is crucial for patient empowerment, leading to improved self-care, adherence to treatment plans, and overall health outcomes. Traditional methods of information delivery, such as written documents and oral communication, often result in poor memorization and comprehension. Recent innovations, such as animation videos, have shown promise in enhancing patient understanding, but comprehensive investigations into their effectiveness across various health care settings are lacking.

**Objective:**

This systematic review aims to investigate the effectiveness of animation videos on health information recall in adult patients across diverse health care sectors, comparing their impact to usual information delivery methods on short-term and long-term recall of health information.

**Methods:**

We conducted systematic searches in PubMed, CINAHL, and Embase databases, supplemented by manual searches of reference lists. Included studies were randomized controlled trials involving adult participants (≥18 years) that focused on the use of animation videos to provide health information measured against usual information delivery practice. There were no language restrictions. Out of 2 independent reviewers screened studies, extracted data, and assessed the risk of bias using the Revised Cochrane risk-of-bias tool for randomized trials (RoB2), Covidence was used to handle screening and risk of bias process. A narrative synthesis approach was applied to present results.

**Results:**

A total of 15 randomized controlled trials—3 in the United States, 2 in France, 2 in Australia, 2 in Canada, and 1 in the United Kingdom, Japan, Singapore, Brazil, Austria, and Türkiye, respectively—met the inclusion criteria, encompassing 2,454 patients across various health care settings. The majority of studies (11/15, 73%) reported statistically significant improvements in health information recall when animation videos were used, compared with usual care. Animation videos ranged from 1 to 15 minutes in duration with the most common length ranging from 1 to 8 minutes (10/15) and used various styles including 2D cartoons, 3D computers, and whiteboard animations. Most studies (12/15) assessed information recall immediately after intervention, with only 3 studies including longer follow-up periods. Most studies exhibited some concerns related to the risk of bias, particularly in domains related to deviations from intended interventions and selection of reported results.

**Conclusions:**

Animation videos appear to significantly improve short-term recall of health information among adult patients across various health care settings compared with usual care. This suggests that animation videos could be a valuable tool for informing patients in different health care settings. However, further research is needed to explore the long-term efficacy of these interventions, their impact on diverse populations, and how different animation styles might affect information recall. Future studies should also address methodological limitations identified in current research, including the use of validated outcome measures and longer follow-up periods.

**Trial Registration:**

PROSPERO CRD42022380016; http://crd.york.ac.uk/prospero/display_record.php?RecordID=380016

## Introduction

Accessible, accurate, and understandable health information is crucial in health care, empowering patients to make informed decisions, take appropriate actions, and actively participate in their own health management [1-3]. It enables patients to understand their conditions, treatments, and health behaviors, leading to improved self-care, better adherence to treatment plans, and overall better health outcomes [4-6].

Studies have shown that lacking or inaccurate knowledge of health conditions or treatment reduces the ability of the patient to engage in treatment and preventive management behaviors [7-9] with lower compliance leading to poorer health outcomes, such as physical disabilities and decreased mental health [4,10].

Information about illness and relevant treatments is traditionally provided through written documents, for example, information pamphlets handed out by a health care professional, or oral information given during a consultation. However, these types of information often lead to poor memorization and comprehension, resulting in potential mistrust and confusion. Furthermore, these types of information were strongly correlated with level of education and health literacy levels [11,12].

Over the past decade, there has been a growing interest in using innovative tools to improve patient information, such as animation videos, to enhance patients’ knowledge and skills in managing their illnesses. Combining verbal and pictorial information in a coordinated way has been shown to enhance learning outcomes, for individuals with high and low health literacy [13-15].

While previous research has delved into the use of animated videos and shown promising results in selected health areas [15], a notable gap exists in the comprehensive examination of animations’ precise influence as a tool for improving the recall of health-related information in general. More knowledge is particularly needed when exploring more technical parameters when using animation videos [16,17]. Factors such as the type of animation video used, the specific health topics addressed, the duration of the animation’s impact, and other relevant variables need to be further investigated.

Therefore, the objective of this systematic review is to investigate the effectiveness of animation videos on health information recall in patients as they engage with the health care system across diverse health care sectors.

## Methods

### Protocol and Registration

The review protocol was registered at PROSPERO (International Prospective Register of Systematic Reviews; ID: CRD42022380016; November 2022). PRISMA (Preferred Reporting Items for Systematic Reviews and Meta-Analyses) guidelines were followed when reporting the search results [18] (Multimedia Appendix 1). Our original protocol specified the inclusion of studies investigating only patients with musculoskeletal disorders. This criterion was later broadened to include studies examining all types of disorders.

### Eligibility Criteria

#### Participants

This systematic review included studies involving adult participants aged 18 years and older in a health care setting. No restrictions were imposed based on ethnicity, gender, or socioeconomic status. However, studies on patients with personality disorders or conditions known to affect cognitive functionality were excluded.

#### Intervention

**Figure 1:**
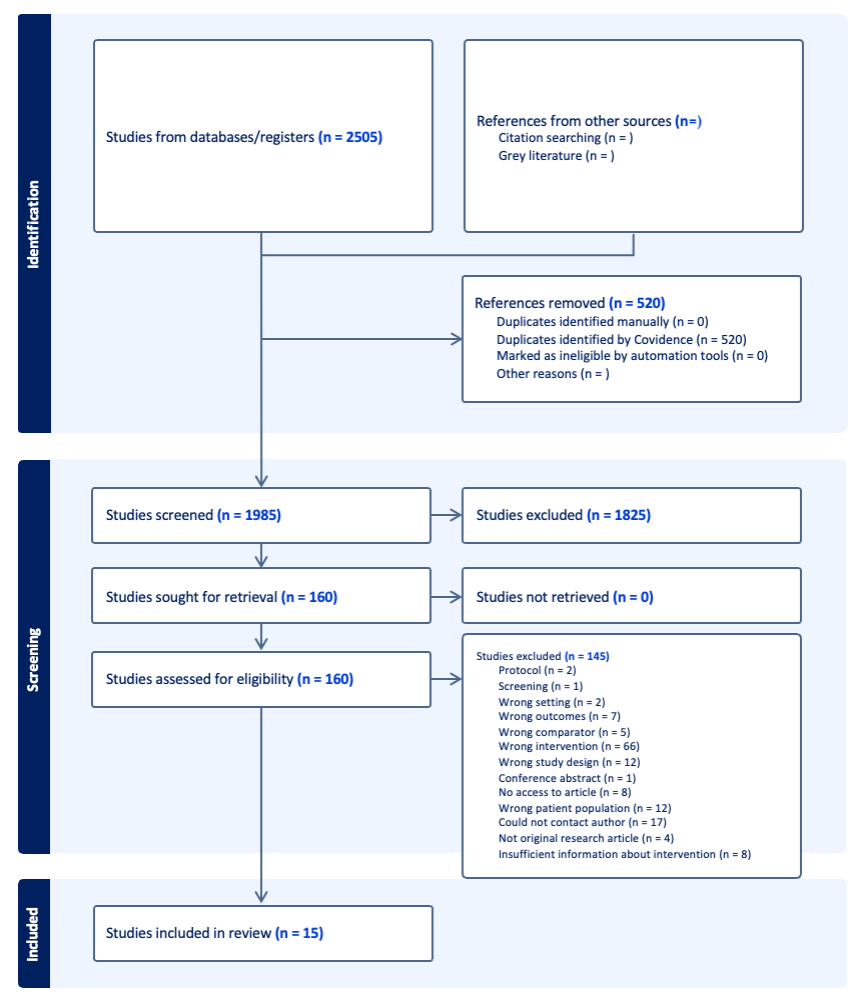
PRISMA (Preferred Reporting Items for Systematic Reviews and Meta-Analyses) flow diagram.

Animation videos were only considered if they were objective and factual and provided information about a patient’s specific health condition, such as the course of treatment or physiological and anatomical facts. In addition, the animation videos should enhance patients’ understanding of their health condition and how to manage it. The delivery mode of interventions should involve either the use of CD-ROMs, websites, tablet devices, computers, or mobile phones within a health care context. The animation was eligible if it consisted of, for example, cartoons, avatars, “whiteboard” animation, or animated 2D or 3D models. The intervention was only eligible if sufficient details about the animation video were provided, either by providing detailed descriptions, screenshots, or links; authors were contacted for more information if details were scarce. We used the exclusion definition of insufficient details about the intervention when it was unclear if the animation video component was the main component in the intervention or just a bi-part of the intervention as a whole. Furthermore, we used the definition could not contact the author if we were uncertain if the studies included what we defined as animation or not, and did not get any response when trying to contact authors to provide sufficient details about the animation component. All exclusion reasoning is displayed in Figure 1.

#### Comparator

Usual care encompasses conventional information delivered by health care professionals, such as the use of written printed materials or verbal communication.

#### Outcome

Health information recall refers to the participant’s ability to remember and recall the information provided by the animation video immediately after receiving it or during a follow-up period. Therefore, all studies that aimed to measure information recall, knowledge gain, or acquisition of information about the specific disorders as a primary or secondary outcome were included.

#### Types of Studies

This review included randomized controlled trials (RCTs) that were peer-reviewed. No specific criteria were set for the language used in the studies. If necessary, a translation tool (DeepL) was used to review and assess a study. The time frame was not restricted, as it was anticipated that research related to animation videos would primarily be limited to the last few decades.

### Search Strategy

A 3-step search was performed with the assistance of an experienced research librarian. First, an initial search was conducted and was ongoing from July 2022 to August 2022. This search was limited to PubMed, CINAHL, and Embase using preliminary subject headings and keywords based on experience and knowledge of the field. Articles that appeared appropriate to the aim were viewed. Notes were made of relevant keywords contained in the title, abstract, and index terms in each relevant article.

The preliminary subject headings and keywords were revised in accordance with the findings obtained in the initial search. Articles found relevant in the first search were set aside to confirm that the second search identified these. 

The second search was performed between September 12, 2022, and September 16, 2022, using the revised subject headings and keywords in PubMed, CINAHL, Embase, and Web of Science. The search was divided into blocks consisting of main keywords and additional variables and is shown in Multimedia Appendix 2. The third and final search was performed under and after the screening process from January 24, 2023, to September 18, 2023, and was conducted using reference lists. Reference lists were manually consulted to identify any additional studies, and finally, a crosscheck was done to confirm that previously identified articles were included in the search.

### Study Selection

A 3-step selection and assessment process were conducted. First, all studies were imported into Covidence, a web-based collaboration software for managing systematic reviews and also to remove duplicates [19].

Second, all titles and abstracts were independently screened twice by 2 reviewers (SH, TSJ, AMS, and MTH). Articles identified for full-text underwent a similar process, being independently screened twice by the 2 reviewers (SH, TSJ, AMS, and MTH). Discrepancies between the reviewers’ decisions in the inclusion and exclusion process were clarified by involving a third reviewer who had not been part of the initial review (SH, TSJ, AMS, and MTH; Figure 1). Finally, all studies reviewed in full-text that did not meet the inclusion criteria were excluded.

### Data Collection

Characteristics and outcomes of the studies were extracted using a data collection form developed in Covidence [19]. This was done by 2 separate reviewers (SH, TSJ, AMS, and MTH), and in case of conflicting understandings, the subject was resolved by discussion or handled by the third reviewer. Data were extracted on author and date, setting (health care sector) and country, sample size, mean age of participants, intervention and comparator, follow-up period, and outcome, including type of measurement instrument.

### Study Quality Assessment

The studies that met the eligibility criteria were assessed for their methodological validity using the Revised Cochrane risk-of-bias tool for randomized trials (RoB2) template [20]. A total of 2 independent reviewers (SH, TSJ, AMS, and MTH) appraised the studies, and in cases of disagreement, a third reviewer was consulted.

### Data Analysis

Due to the diverse nature, use of measurement instruments, and the heterogeneity of articles in this field, it was not planned to perform a meta-analysis. Therefore, a narrative synthesis approach was planned. Detailed information on the study characteristics, and methodological quality, as well as a summary of the outcome measures and the results, was obtained. Also, the narrative synthesis summarized the findings according to three pre-identified outcome-related categories (length of the animation videos and time for outcome assessment; animation styles and health-related topics; and comparators and the role of the animation video) inspired by the data extraction template made for this study. We have used *P*<.05 as an indicator of effect.

## Results

### Study Selection

A total of 2505 studies were identified, yielding 79% (1985/2505) studies after removal of duplicates. After reviewing the 1985 titles and abstracts, 1825 studies were excluded, resulting in a total of 160 (8.1%) eligible for full-text assessment. After full-text assessment, 145 additional studies were excluded, resulting in a total of 15 included studies (Figure 1). All inconsistencies in data assessment and extraction were resolved by consensus among the reviewers. A total of 2454 patients were included, and details of the study selection are displayed in Figure 1.

### Study Characteristics

Detailed characteristics and results of the included studies are shown in Multimedia Appendix 3. All studies were conducted in the last 10 years, with most studies 75% (10/15) published within the past 5 years [21-30]. Of the 15 studies included the majority 12 (80%) were conducted in high-income countries [21,22,25-27,29-35], and 3 (20%) were conducted in upper- to middle-income countries [23,24,28]. Most studies originated from the United States (20%) [21,26,29], Australia (13.3%) [32,33], Canada (13.3%) [27,34], and France (13.3%) [22,30], and one (6.7%) each from Japan [31], Austria [35], United Kingdom [25], Singapore [24], Brazil [23], and Türkiye [28].

### Length of the Animation Videos and Time for Outcome Assessment

The animation videos exhibited a diverse range of durations, spanning from 1 to 15 minutes. Dividing the length into percentiles revealed distinct patterns among the studies. A third (5/15) of the studies used short animation videos lasting 1 to 5 minutes [24,26,30,31,34]. An equal proportion featured middle-length animation videos ranging from 6 to 8 minutes [21,23,25,27,29]. In total, 3 studies used longer animation videos, falling within the 13- to 15-minute range [22,32,33]. In addition, 2/15 had animation videos with unspecified durations [28,35]. Most of the studies (12/15) assessed the outcome immediately after the patients were given the intervention [22-24,26,27,29-35] or on the day of treatment [25]. Only 3 of 15 studies had follow-up, one after 1 month [28], one after 6 weeks [32], and one after 6 and 8 weeks [21].

### Animation Styles and Health-Related Topics

The studies used a variety of animation styles on different health-related topics. 2D cartoon animated videos were used in 7 studies, on the subjects of diabetes food care [28]; imaging and inevitable consequences of lower back pain [23]; living with atrial fibrillation [27]; anesthesia during surgery [31]; the etiology, symptoms, and treatments for stress and urgency urinary incontinence [21]; and preoperative retention undergoing bowel resection [33] and postoperative risk with benign parotid tumor [22]. 3D computer animation was used in 4 studies, with topics regarding; informed consent before operative laparoscopy [32]; informed content before elective inpatient coronary angiography [30]; knowledge regarding Mohs surgery [25]; and age-related cataract surgery [35]. A total of 3 studies used narrated whiteboard animation the subjects presented by this form of animation were opioid safety and proper usage, storage, and disposal [29]; first-time intravenous fluorescein angiography [34]; and patients undergoing coronary angiography [24]. Finally, one study used a mixture of 2D and 3D animation on the subject of Mohs surgery [26].

### Comparators and the Role of the Animation Video

A variety of comparators, commonly referred to as “usual care,” were used across the studies. Notably, the majority of the studies (12/15) evaluated animation videos as a supplementary tool alongside usual care. Among these 12 studies, 6 used usual care that combined written materials with verbal information provided by health care professionals [22,24,25,29,30,35]. Out of 4 other studies within this subset described usual care as either a routine verbal consent process [26,32] or individual face-to-face conversations [27,31]. A more flexible approach to usual care was noted in 1 study, which incorporated a mix of verbal explanations, drawings, or picture imagery [21]. The remaining study relied solely on written information sheets as usual care [33]. In contrast, the last 3 studies out of the 15 had a different approach, examining animation videos as an alternative, rather than a supplement, to their usual care. In total, 2 of these studies used a combination of verbal and written information as their usual care [28,34] while the last study only used written materials [23].

### Outcomes

#### Health Information Recall

The 15 studies used different outcome measurements to assess health information recall. All the studies used self-developed questionnaires, which were based on the information provided during the consultation. The knowledge measurement tools included multiple-choice answers, open-ended questions, and statement questions with true or false and yes or no options. The studies included in the review used varying numbers of questions related to the specific medical subject, with the number of questions ranging from 4 to 16.

#### Positive Effects Using Animation Videos

In total, 11 studies reported statistically significant improvements in health information recall when compared with control groups [22,24,26-32,34,35].

Notably when looking at animation style, the 3 studies that used whiteboard animation as an educational tool demonstrated a significant positive effect over usual care [24,29,34]. A total of 2 out of the 3 studies using 3D animation videos had a positive effect [32,35], and 5 of the total 8 studies using 2D cartoon animation [22,27,28,31] including the study using a mixture of 2D and 3D [26] had a positive effect.

When looking at the time of outcome assessment 10 out of the 11 studies that found effect was measured immediately after intervention [22,24,26,27,29-32,34,35]. One of those studies did not have a persistent effect at the 6-week follow-up assessment [32]. Conversely, the study conducted by Dincer and Bahçecik [28] in 2021 observed a positive effect 4 weeks post intervention [28].

#### Equal Effect of Using Animation Video

In total, 3 out of the 15 studies found equal benefits between the use of animation video and usual care [21,25,33]. Two of the studies used 2D cartoon animation [21,33] and 1 used a 3D animation video [25]. In the cases of these 3 studies, the usual care alone achieved the same positive effect as supplementing usual care with the animation video. The studies’ usual care consisted of leaflets and verbal information [25], a combination of verbal communication, drawing or pictorial imagery, using a more open and flexible information style [21], and finally a simple information sheet [33].

#### Negative Effect of Using Animation Video

In 1 study [23], it was observed that usual care achieved significantly greater benefits than the use of animation videos when assessing the accuracy of patients’ responses regarding the correct use of imaging. Notably, the animation video was introduced as an alternative to conventional consent information. The comparators to animation were in this case (1) a 2-page written summary on an A4 paper and (2) an infographic solution combining both imagery and written summary, both these solutions had equal positive effects.

### Study Quality Assessment

**Figure 2:**
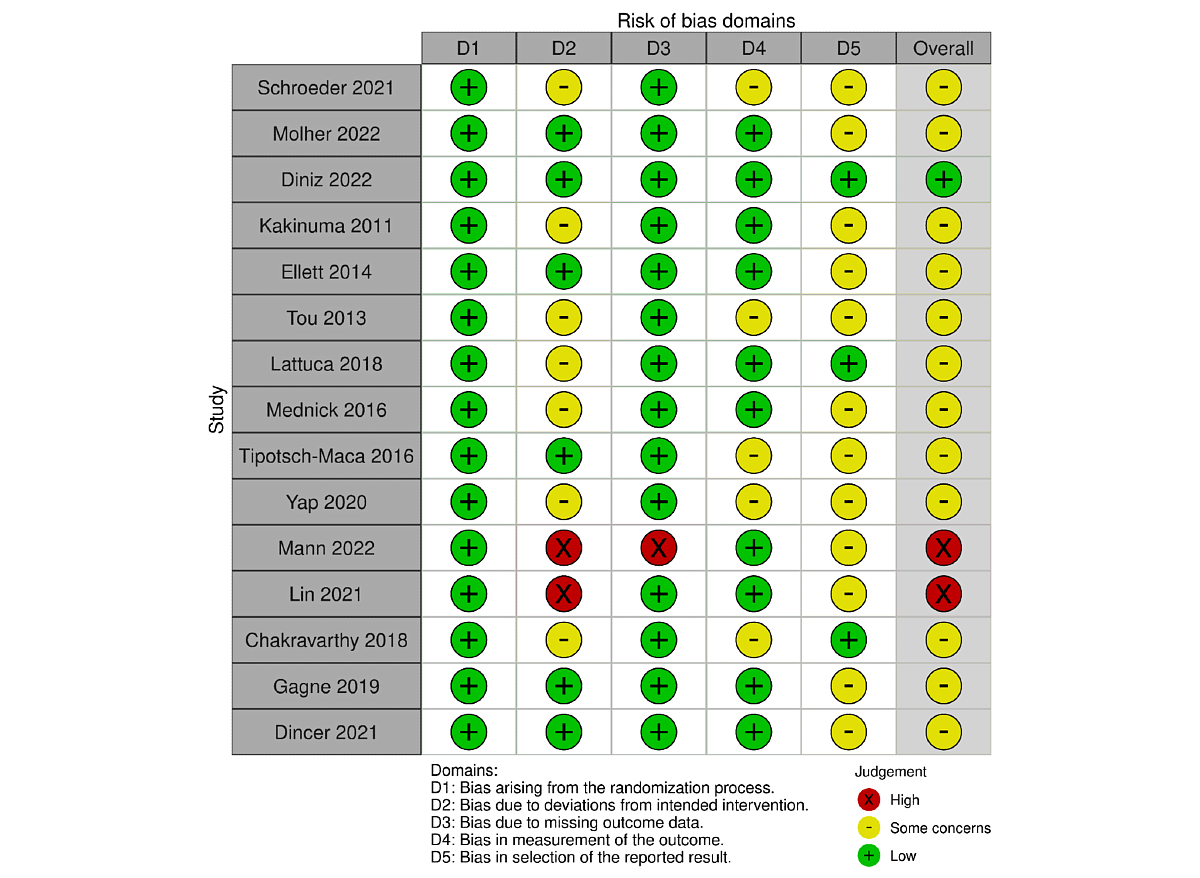
Risk of bias of included studies in this review of the effectiveness of animation as a tool for recall of health information, Denmark 2022-2024 [21-35].

An overview of the risk of bias assessment for the included studies is presented in Figure 2 [21-35]. In general, most studies exhibited “some concerns” related to the risk of bias, with the most prominent issues being observed in domain D2: Bias due to deviations from intended interventions. The primary reason for this was our inability to access trial protocols or get a sufficient description of the protocol, which would have provided crucial insights into the trial context. Without access or information in the protocols, it was difficult to determine if the intended interventions were consistently followed, which could have potentially introduced bias into the results. In some cases, there was a lack of transparency regarding blinding procedures, further contributing to concerns in this domain.

Moreover, the domain “D5: Bias in selection of the reported results,” also raised some concerns. These issues were partly due to the unavailability of information on study protocols, making it difficult to determine if the reported results align with a prespecified analysis plan.

To a lesser extent, some concerns were identified in the domain “D4: Risk of bias in the measurement of the outcome.” Many studies of interventions in this area face a common challenge of using self-developed outcome measurement tools instead of validated instruments. This is because the outcomes being measured are often highly specific and directly related to the particular disorder targeted by the animation. However, using nonvalidated tools raises questions about the reliability and validity of the outcome measurements.

## Discussion

### Principal Findings

The primary objective of this systematic review was to investigate the effectiveness of animation videos as informative tools in the health care system, particularly in enhancing patient health information recall. The main finding is that 11 of the 15 studies reported a significant positive impact of animation videos on health information recall. The studies predominantly originated from high-income countries and were mostly conducted within the last decade, with a significant concentration (75%) published in the past 5 years. The studies demonstrated a diversity in the length and style of animation videos, ranging from short (1-5 minutes) to longer durations (up to 15 minutes). Different animation styles were used, including 2D cartoon animation, 3D computer animation, and narrated whiteboard animation. This variety underscores the adaptable nature of animation as a medium for conveying health information.

### Comparison to Previous Work

Similar results are found in a recent systematic review investigating the effectiveness of animations as an information medium for patients and the general public; they found that across 30 trials assessing knowledge, 19 of those had greater knowledge compared with a range of comparators. However, the evidence base was highly variable, with mostly small trials [36]. The same tendencies are shown in a systematic review and meta-analysis by Feeley et al [37], which found an overall weighted effect size of 0.38, which indicates that improvements are modest, but not less reliable across 20 out of 21 included studies [37]. This indicates an overall positive effect in increasing patient understanding when using animation videos across different health care settings.

Contrasting our work with the review by Moe-Byrne et al [36], several distinctions become apparent. First, our review focuses exclusively on animations designed as a tool for patient information, whereas the review by Moe-Byrne et al [36] includes public interventions, such as health promotion campaigns. Second, we considered solely RCTs, while the previous review also encompassed quasi-experimental studies. Finally, their analysis was not limited to health information recall but also examined changes in attitudes and behaviors [36]. Interestingly, Feeley et al [37] found that animations work better with patients than they do with samples from the general population or mixed groups of patients and nonpatients [37]. This finding supports our focus on patient populations and suggests that animations tailored specifically for certain patient groups may be particularly effective, possibly due to increased motivation and relevance of the information presented.

Notably, in our systematic review, all studies using whiteboard animation showed a significant positive effect, suggesting its effectiveness as an educational tool [25,29,34]. Even though only 3 studies used whiteboard animation, this can indicate that simple forms of animations are efficient in their ability to inform patients. This finding aligns with previous studies investigating the impact of details in animation videos, and their ability to be informing. For example, it was found that realism in animation, such as the level of visual detail, did not significantly affect cognitive learning performance, suggesting different levels of animation details can be equally effective for instructional purposes [38]. Furthermore, a study found that increasing detail can result in overloading the viewer’s visual information processing, resulting in worse performance tests regarding information retention [39].

Most studies assessed recall immediately after intervention [22-24,26,27,29-35], with only a few extending the follow-up period [21,28,32]. This raises questions about the long-term efficacy of animations in patient recall since only 3 out of the 15 studies had follow-up, and only one of them found a significant effect at 4 weeks [28]. When comparing these results with the systematic review by Moe-Byrne et al [36] they conclude similar findings, indicating that the effect tends to be most present immediately after intervention [36], suggesting that while animations are effective in the short term, the sustainability of this effect warrants further investigation.

Our systematic review observed a diverse range of animation durations, from 1 to 15 minutes. Interestingly, Feeley et al [37] found that video length was a significant moderate of effectiveness, but only at an α=.06 criterion. Their analysis suggested that longer videos yielded smaller learning effects across the 16 studies that reported video length [37]. This aligns with our observation that a third of our included studies used short animation videos lasting 1 to 5 minutes, all of which demonstrated positive effects. These findings collectively suggest that concise, well-designed animations might be more effective in conveying health information, possibly due to their ability to maintain viewer attention and avoid cognitive overload.

Finally, our review found that most studies (80%) were conducted in high-income countries, Feeley et al [37] reported that 9 out of 21 studies were conducted in the United States, with the remaining 12 done in countries Germany, Japan, the Netherlands, Australia, Taiwan, Singapore, and Saudi Arabia. This similarity in geographical distribution underscores the need for more research in diverse global settings, particularly in low- and middle-income countries.

### Strengths and Limitations

For this systematic review, we used different processes to reduce the potential risk of bias. First, it was a strength that the review protocol was registered with PROSPERO, and the PRISMA guidelines were adhered to, ensuring transparency and consistency in our reporting. Our collaboration with an experienced research librarian enabled a thorough 3-step search across multiple databases and manual searches, enhancing the breadth and relevance of the literature covered. The use of dual independent reviewers for screening, selection, data extraction, and risk of bias assessment, along with the systematic use of Covidence software, helped minimize biases and ensured a rigorous selection process. Using the Revised Cochrane risk-of-bias tool for randomized trials added robustness to our methodology, with discrepancies resolved through consultation with a third researcher.

This systematic review had several limitations. First, our search strategy may have missed some relevant papers. While we conducted a comprehensive search across multiple databases (PubMed, CINAHL, and Embase) and performed manual searches of reference lists, we did not have access to all potentially relevant databases, such as PsycINFO or CENTRAL. To mitigate this, we worked closely with an experienced research librarian to develop and refine our search strategy. However, future reviews could expand the search to include additional databases and consider using a wider range of search terms to capture all relevant studies.

Second, we faced challenges regarding the accessibility and evaluation of the animation video content used in the included studies. Only 8 out of 15 studies provided direct links to the animation videos, while the remaining studies offered only screenshots or written explanations. This limited our ability to fully assess the quality and effectiveness of the animations. Furthermore, we had to exclude several studies from the screening process due to this problem. To address this, we attempted to contact all authors to gain access to the animation videos or sufficient information about the intervention, but with limited success. This limitation may have influenced our results by preventing a comprehensive analysis of the animation characteristics that contribute to effective health information recall. Future studies in this area should focus on elaborating the design of the animation video and its role in the intervention.

Third, our decision to include only RCTs in this systematic review may have limited the breadth of evidence we were able to consider. While this choice was made to focus on the highest quality evidence available, it potentially excluded valuable insights from well-designed quasi-experimental studies or other study types. To mitigate this, we ensured a thorough search and screening process for RCTs. However, this limitation may have influenced our results by potentially overestimating the effectiveness of animation videos, as RCTs with positive outcomes are more likely to be published. In addition, we may have missed nuanced findings from real-world implementations that are often captured in quasi-experimental designs. For future reviews in this area, researchers could consider including a wider range of study designs, such as high-quality quasi-experimental studies, while using appropriate tools to assess their risk of bias. This approach could provide a more comprehensive understanding of the effectiveness of animation videos in various real-world health care settings, while still maintaining a focus on methodological rigor. Finally, the final search was conducted in mid-2023 and additional studies may have been published in the interim given the progressive nature of this topic.

### Future Directions

First, our review and Feeley et al [37] suggest that shorter animations may be more effective. Future interventions should focus on creating concise, targeted animations, typically around 5-8 minutes in length, to maintain viewer engagement and minimize cognitive overload. Second, the effectiveness of whiteboard animations in our review suggests that simple, clear animations can be highly effective. Future work should explore how to maximize the impact of these simpler animation styles across various health topics.

Third, given the limited long-term follow-up in current studies, future interventions should incorporate strategies to reinforce information over time. This could include providing patients with access to animations for repeated viewing or developing complementary materials that build on the animated content. Our systematic review is a great stepping stone for organizations, institutions, and so on, that want to develop their own animation videos on the subject of health care information.

### Conclusion

In conclusion, this systematic review finds an overall positive impact of animation videos on short-term health information recall. Further studies should investigate the identified research gaps, particularly the long-term efficacy, impact on diverse populations, and the impact of animation style on the effectiveness of health information recall. Furthermore, future studies should focus on including a detailed description of the type of animation used in the intervention, or provide a link, to enable full transparency and usability of the studies.

## Appendix

Multimedia Appendix 1 PRISMA checklist.

Multimedia Appendix 2 Search Strategy.

Multimedia Appendix 3 Overview/characteristics of included studies.

## Abbreviations

PRISMA: Preferred Reporting Items for Systematic Reviews and Meta-Analyses
PROSPERO: International Prospective Register of Systematic Reviews
RCT: randomized controlled trial
RoB2: Revised Cochrane risk-of-bias tool for randomized trials
