# Supplementary material for: The Effectiveness of Video Animations as a Tool to Improve Health Information Recall for Patients: Systematic Review
Source: J Med Internet Res. 2024 Dec 30;26:e58306. doi: 10.2196/58306 (PMC11730234; doi:10.2196/58306)
Supplement: Multimedia Appendix 2 [file jmir_v26i1e58306_app2.pdf]

Appendix 2: Search Strategy

Pubmed

((("Multimedia"[MeSH Terms] OR "Cartoons as Topic"[MeSH Terms] OR "Motion Pictures"[MeSH Terms] OR "Multimedia"[Text Word] OR "animation*"[Text Word] OR "motion graphic"[Text Word] OR "motion graphics"[Text Word] OR "cartoon*"[Text Word] OR "portable video*"[Text Word] OR "medical illustration"[Text Word] OR "medical illustrations"[Text Word] OR "audiovisual method"[Text Word] OR "audiovisual methods"[Text Word] OR "video*"[Title]) AND ("Patient Education as Topic"[MeSH Terms] OR "Consumer Health Information"[MeSH Terms] OR "Information Literacy"[MeSH Terms] OR "Health Communication"[MeSH Terms] OR "health education"[Text Word] OR "patient education"[Text Word] OR "health literacy"[Text Word] OR "ehealth literacy"[Text Word] OR "e health literacy"[Text Word] OR "Consumer Health Information"[Text Word] OR "communicat*"[Title] OR "educat*"[Title] OR "information*"[Title]) AND ((("health information"[All Fields] AND "recall"[Text Word]) OR "health knowledge"[Text Word] OR "acquisition of health"[Text Word] OR (("retention, psychology"[MeSH Terms] OR ("retention"[All Fields] AND "psychology"[All Fields]) OR "psychology retention"[All Fields] OR "retention"[All Fields] OR "retentions"[All Fields] OR "retentive"[All Fields] OR "retentiveness"[All Fields]) AND "health information"[Text Word]) OR (("retention, psychology"[MeSH Terms] OR ("retention"[All Fields] AND "psychology"[All Fields]) OR "psychology retention"[All Fields] OR "retention"[All Fields] OR "retentions"[All Fields] OR "retentive"[All Fields] OR "retentiveness"[All Fields]) AND "health education"[Text Word]) OR (("health education"[Text Word] OR "health information"[All Fields]) AND "effectiveness"[Text Word]) OR (("health education"[Text Word] OR "health information"[All Fields]) AND "acquisition"[Text Word]) OR "health knowledge, attitudes, practice"[MeSH Terms]) OR (effectiveness[Title] OR improv*[Title] OR approach[Title] OR usefulness[Title] OR effect[Title] OR assessing[Title] OR benefits[Title] OR motivat*[Title]))) AND (("randomized controlled trial"[Publication Type] OR "controlled clinical trial"[Publication Type] OR "randomized"[Title/Abstract] OR "placebo"[Title/Abstract] OR "drug therapy"[MeSH Subheading] OR "randomly"[Title/Abstract] OR "trial"[Title/Abstract] OR "groups"[Title/Abstract]) NOT ("animals"[MeSH Terms] NOT "humans"[MeSH Terms])))
Records retrieved: 771

Cinahl

NO.	Searches
1	(MH “(Multimedia”)
2	(MH “Audiovisuals+”)
3	TI (multimedia or animation or motion graphic* or cartoon or portable video* or medical illustration* or audiovisual method* or video) OR AB (multimedia or animation or motion graphic* or cartoon or portable video* or medical illustration* or audiovisual method*)
4	S1 OR S2 OR S3
5	(MH “Health Education+”)
6	(MH “Health information+”)
7	(MH “Information Literacy+”)
8	(MH “Health Promotion+”)
9	(TI (Health communication or health education or patient education or health promotion or health information or communicat* or educat* or information*) OR AB (Health communication or health education or patient education or health promotion or health literacy or ehealth literacy or consumer health information)
10	S5 OR S6 OR S7 OR S8 OR S9
11	TI ((health education or health information or health knowledge= AND (recall or retention or retentive* or acquisition or effect* or improve* or approach or usefulness or assessing or benefit*)) OR AB ((health education or health information or health knowledge) AND (recall or retention or retentive* or acquisition or effect* or improve * or approach or usefulness or assessing or benefit*))

12	(MH “Memory+”)
13	(MH “Health Knowledge”)
14	S11 OR S12 OR S13
15	S4 AND S10 AND S14
16	(MH “Double-Blind Studies”) OR (MH “Single-Blind Studies”) OR (MH “Randomized Controlled Trails+”)
17	(MH “Random Assignment”)
18	(MH “Pretest-Posttest Design+”)
19	(MH “Cluster Sample+”)
20	TI randomised OR randomized
21	AB random*
22	TI trial
23	MH sample size AND AB (assigned OR allocated OR control)
24	(MH “Placebos”)
25	PT randomized controlled trial
26	AB control W5 group
27	MH crossover design OR MH comparative studies
28	AB cluster W3 RCT
29	(MH “animals+”)
30	MH animal studies
31	TI animal model*
32	S29 OR S30 OR S31
33	MH human
34	S32 NOT S33
35	S16 OR S17 OR S18 OR S19 OR S20 OR S21 OR S22 OR S23 OR S24 OR S25 OR S26 OR S27 OR S28
36	S35 NOT S34
37	S4 AND S10 AND S14 AND S36

Records retrieved: 576

Web of Science

1	Animation (Topic)
2	Cartoon* (Topic)
3	Motion graphics (Topic)
4	Motion picture* (Topic)
5	Multimedia (Topic)
6	Portable video* (Topic)
7	Medical illustration* (topic)
8	Video (Title)
9	#1 OR #2 OR #3 OR #4 OR #5 OR #6 OR #7 OR #8
10	Health education (Topic)
11	Health information (Topic)
12	Information literacy (Topic)
13	Health communication (Topic)

14	Patient education (Topic)
15	Health literacy (Topic)
16	Ehealth literacy (Topic)
17	Consumer health information (Topic)
18	Communicat* (Title) or educat* (Title) or information* (Title)
19	#10 OR #11 OR #12 OR #13 OR #14 OR #15 OR #16 OR #17 OR #18
20	Health education or health information or health knowledge (Topic) and recall or retention or retentive* or acquisition or effect* or improve* or approach or usefulness or assessing or benefit* (Topic)
21	TT S=(randomized or placebo or randomly or trial)) or TI=(trial)
22	#9 AND #19 AND #20 AND #21

Records retrieved: 736

Embase

1	Exp multimedia/
2	Exp videorecording/
3	Exp movie/
4	Animation.ab.ti
5	“cartoon”.ab.ti.
6	Motion graphics.ab.ti.
7	“motion picture*”.ab.ti.
8	Multimedia.ab.ti.
9	“portable video*”.ab.ti.
10	Medical illustration.ab.ti.
11	Video.ti.
12	1 or 2 or 3 or 4 or 5 or 6 or 7 or 8 or 9 or 10 or 11
13	Exp patient education/
14	Exp consumer health information/
15	Health education.ab.ti.
16	Health information.ab.ti.
17	Information literacy.ab.ti.
18	Health communication.ab.ti.
19	Patient education.ab.ti.
20	Health literacy.ab.ti.
21	Ehealth literacy.ab.ti.
22	(communicat* or educat* or information*).ti.
23	Exp health literacy/
24	13 or 14 or 15 or 16 or 17 or 18 or 19 or 20 or 21 or 22 or 23
25	Exp attitude to health/
26	(health education or health information or health knowledge).mp. [mp=title, abstract, heading word, drug trade name, original title, device manufacturer, drug manufacturer, device trade name, keyword heading word, floating subheading word, candidate term word]
27	(recall or retention or rententive* or acquisition or effect* or improve* or approach or usefulness or assessing or benefit*).mp. [mp=title, abstract, heading word, drug trade name,

	original title, device manufacturer, drug manufacturer, device trade name, keyword heading word, floating subheading word, candidate term word]
28	26 AND 27
29	25 OR 28
30	(randomized or placebo or randomly or trial).mp.[mp=title, abstract, heading word, drug trade name, original title, device manufacturer, drug manufacturer, device trade name, keyword heading word, floating subheading word, candidate term word]
31	12 and 24 and 30

Records retrieved: 422
