# Supplementary material for: The Effectiveness of Video Animations as a Tool to Improve Health Information Recall for Patients: Systematic Review
Source: J Med Internet Res. 2024 Dec 30;26:e58306. doi: 10.2196/58306 (PMC11730234; doi:10.2196/58306)
Supplement: Multimedia Appendix 3 [file jmir_v26i1e58306_app3.pdf]

Appendix 3

Authors	Outcome assessment	Total sample size (intervention, control) N (n, n)	Population	Intervention	Comparator	Key results
Schroeder et al. [21]	Immediately after intervention and 6 to 8 weeks	98 (50, 48)	Patients with urinary incontinence	Watched an 7.5minute 2D cartoon animation video undergoing postoperative risk	Received counseling included conversation-based education with the opportunity to ask follow-up questions at the end	Immediate posttest knowledge scores trended toward intervention compared with control, but not significantly. No difference in the delayed posttests. Both education methods demonstrated significant differences between pre-test, immediate posttest, and delayed posttest scores
Molher et al [22]	Immediately after intervention	56 (27, 29)	Patients with benign parotid tumor	Watched a 15-minute 2D cartoon animation video emphasizing	Received information orally and in writing from the surgeon	The intervention group achieved significantly greater

				7 postoperative risks		knowledge scores compared with the control group
Diniz et al [23	Immediately after intervention	159 (53, 53, 53)	Patients with nonspecific LBP[c] of any duration	Intervention group 1: A 6.5minute 2D cartoon animated video presenting different scenarios for managing LBP. Intervention group 2: interactive infographics on A4 paper debunking myths and presenting facts about LBP	Received a 2-page written summary on plain A4 paper detailing LBP diagnosis and treatment recommendations	Intervention group 1 performed significantly worse than intervention group 2 and the control group when looking at knowledge regarding correct use of imaging.
Kakinuma et al [31	1 day after intervention	211 (106, 105)	Patients undergoing cancer surgery	Watched a 2minute 2D cartoon animation explaining anesthesia methods and complications	Received a routine pre-anesthetic interview and risk assessment	The intervention group achieved significantly better knowledge scores compared with a control group

Ellet et al [32	Immediately and 6 weeks after intervention	41 (21, 20)	Patients undergoing pelvic pain-operative laparoscopy	Watched a 15-minute 3D animation undergoing operation procedure, benefits, risks, and hospital process and discharge.	Routine verbal consent process	The intervention group showed significantly increased knowledge scores compared with the control group immediately after the intervention. However, this advantage did not persist after 6 weeks
Tou et al [33]	Immediately after intervention	31 (16, 15)	Patients undergoing colorectal surgery	Watched a 13minute 2D cartoon animation video explaining pre-and postoperative care.	Received an information sheet, including details about the role of the preadmission clinic, bowel preparation, stoma information, bowel surgery, postoperative recovery, catheters, and pain control	There was no significant difference in knowledge scores between the intervention and the control group
Lattuca et al [30]	Immediately after intervention	821 (406, 415)	Patients undergoing coronary angiography	Watched a 5-minute 3D animation video undergoing	Received standardized oral information and a national	The intervention group showed a significantly

				operation procedure, benefits, and risks	standard written form	higher knowledge score compared with the control group consistently observed across age and education level subgroups
Mednick et al [34]	Immediately after intervention	52 (26, 26)	Patients undergoing an initial IVFA[d] investigation	Watched a 3.5minute whiteboard animation video undergoing clinical indications, risks, benefits,and administration	Received IVFA information through standard physician-patient interaction to obtain standard consent	The intervention group achieved significantly higher knowledge mean scores compared with a control group
Tipotsch-Maca et al [35]	Immediately after intervention	123 (59, 64)	Patients undergoing age-related cataract surgery	Watched an 3D animation video undergoing an operation procedure. Length unknown	Received an information brochure followed by a face-to-face discussion with the same physician, covering cataract definition, surgical procedure, alternatives, risks, and	Both groups improved their knowledge, but patients in the intervention group performed significantly better compared with the control group

					advisable postoperative behavior	
Yap et al [24]	Immediately after intervention	332 (252, 80)	Patients undergoing coronary angiography and/or angioplasty	Watched a 3.3minute whiteboard animation video that was related to the procedure, risks, benefits, and post-care	Received routine standard of care, including counseling by the physician and an information sheet about the procedure	The intervention group initially had lower knowledge scores than the control group, but after watching the video, their knowledge scores significantly increased compared with before
Mann et al [25	On the day of surgery	168 (49, 49)	Patients referred to MMS[a]	Watched a 7.3minute 3D animation video demonstrating MMS principles.	Received standard MMS patient education by mail, including information leaflets and verbal descriptions	The intervention group achieved significantly higher knowledge scores than the control group
Lin et al [26]	Immediately after intervention	120 (60, 60)	Patients referred to MMS	Watched an 4.2 minute 2D and 3D animation video explaining anesthesia, surgical stages, the	Received routine verbal consent process	The intervention group demonstrated significantly higher knowledge scores than

				timing of surgery, cure rate, scar, and reconstruction		the control group, and in the subgroup of patients without previous consultation with a Mohs surgeon, the intervention group had significantly higher knowledge scores compared with the control group
Chakravarthy et al [29]	Immediately after intervention	52 (25, 27)	Patients with a chief complaint of pain	Watched a 6-minute whiteboard animation video discharge instructions on opioid safety and proper usage, storage, and disposal	Received verbal discharge instructions from nursing staff and a medication information sheet for opioids	The intervention group demonstrated significantly higher knowledge acquisition compared with the control group
Gagné et al [27]	Immediately after intervention	60 (30, 30)	Patients with AF[b]	Watched an 8-minute 2D cartoon animation video undergoing information about the	Received face-to-face educational session and a 20-page booklet	The intervention group demonstrated a significantly greater increase in

				heart, types of AF, risks, symptoms, and signs of stroke		knowledge compared with control group
Dincer et al [28]	1 month after intervention	130 (65, 65)	Patients with diabetes	Watched a 2D cartoon animation video containing basic information required for successful foot care. Length unknown	Received education in diabetic foot care in line with clinical guidelines in the hospital diabetes education room	The intervention group showed significantly higher knowledge scores compared with the control group

[a]MMS: Mohs micrographic surgery.

[b]AF: atrial fibrillation.

[c]LBP: lower back pain.

[d]IVFA: Intravenous fluorescein angiography.
